# A Biosafety Study of Human Umbilical Cord Blood Mononuclear Cells Transduced with Adenoviral Vector Carrying Human Vascular Endothelial Growth Factor cDNA In Vitro

**DOI:** 10.3390/biomedicines11072020

**Published:** 2023-07-18

**Authors:** Ilnur I. Salafutdinov, Dilara Z. Gatina, Maria I. Markelova, Ekaterina E. Garanina, Sergey Yu. Malanin, Ilnaz M. Gazizov, Andrei A. Izmailov, Albert A. Rizvanov, Rustem R. Islamov, András Palotás, Zufar Z. Safiullov

**Affiliations:** 1 Department of Histology, Cytology and Embryology, Kazan State Medical University, Kazan 420012, Russia; ilnazaziz@mail.ru (I.M.G.); gostev.andrei@gmail.com (A.A.I.); rustem.islamov@gmail.com (R.R.I.); redblackwhite@mail.ru (Z.Z.S.); 2 Institute of Fundamental Medicine and Biology, Kazan Federal University, Kazan 420008, Russiasergen83@mail.ru (S.Y.M.); rizvanov@gmail.com (A.A.R.); palotas@asklepios-med.eu (A.P.); 3 Asklepios-Med (Private Medical Practice and Research Center), H-6722 Szeged, Hungary; 4 Tokaj-Hegyalja University, H-3910 Tokaj, Hungary

**Keywords:** cord blood stem cell transplantation, genetic vectors, vascular endothelial growth factor A, transcriptome, secretome, biosafety, genetic therapy

## Abstract

The biosafety of gene therapy remains a crucial issue for both the direct and cell-mediated delivery of recombinant cDNA encoding biologically active molecules for the pathogenetic correction of congenital or acquired disorders. The diversity of vector systems and cell carriers for the delivery of therapeutic genes revealed the difficulty of developing and implementing a safe and effective drug containing artificial genetic material for the treatment of human diseases in practical medicine. Therefore, in this study we assessed changes in the transcriptome and secretome of umbilical cord blood mononuclear cells (UCB-MCs) genetically modified using adenoviral vector (Ad5) carrying cDNA encoding human vascular endothelial growth factor (VEGF165) or reporter green fluorescent protein (GFP). A preliminary analysis of UCB-MCs transduced with Ad5-VEGF165 and Ad5-GFP with MOI of 10 showed efficient transgene expression in gene-modified UCB-MCs at mRNA and protein levels. The whole transcriptome sequencing of native UCB-MCs, UCB-MC+Ad5-VEGF165, and UCB-MC+Ad5-GFP demonstrated individual sample variability rather than the effect of Ad5 or the expression of recombinant *vegf165* on UCB-MC transcriptomes. A multiplex secretome analysis indicated that neither the transduction of UCB-MCs with Ad5-GFP nor with Ad5-VEGF165 affects the secretion of the studied cytokines, chemokines, and growth factors by gene-modified cells. Here, we show that UCB-MCs transduced with Ad5 carrying cDNA encoding human VEGF165 efficiently express transgenes and preserve transcriptome and secretome patterns. This data demonstrates the biosafety of using UCB-MCs as cell carriers of therapeutic genes.

## 1. Introduction

Gene therapy is an actively developing area in practical medicine not only for the correction of inherited diseases [1,2], but also in regenerative medicine to activate endogenous tissue potential [3]. In vivo or direct gene therapy is based on the delivery of therapeutic genes with plasmid or viral vectors, which is predominantly systemic in nature and involves the transduction of different cells in a variety of body organs [2]. Ex vivo or cell-mediated gene therapy for the delivery of transgenes employs stem or mature cells of autogenous or allogenic origin [4]. Ex vivo gene therapy excludes the direct effect of vector antigens with a host immune system and provides temporary or permanent expression of transgenes by genetically modified cells. The rationale for the use of a particular gene therapy depends on the pathogenetic aspects of the disease [5]. However, the biosafety of direct and cell-mediated gene delivery strategies remains a critical issue in translating gene therapy potentiality from preclinical studies to clinical trials. The side effects of vector systems on the recipient organism mainly pertain to immunogenicity and mutagenicity [6], whereas little is known about the effects of transgene expression on transduced cells.

Advances in gene therapy are opening up new perspectives in the treatment of central nervous system (CNS) diseases. The natural limits of CNS regeneration pose major problems for the treatment of neurological disorders of various aetiologies [7]. The delivery of therapeutic genes encoding neurotrophic factors to the brain and spinal cord of patients with neurodegenerative diseases, traumatic injuries, or ischaemic stroke is a prospective approach to increase neuronal survival in the acute phase, as well as to stimulate directed axonal growth, remyelination, and the recovery of lost interneuronal connections in the rehabilitation period [8]. However, in clinical practice worldwide, there are no effective neuroregenerative therapies available for these patients, and symptomatic treatment has no effect on quality of life or life expectancy [9,10].

Cell-mediated gene therapy is effectively used to treat hereditary diseases. Severe combined immunodeficiency was the first hereditary disease for which the use of recombinant cDNA encoding a protein capable of restoring lost lymphocyte function was proposed for treatment. This treatment was proposed in 1990 [11]. To deliver the therapeutic gene to mature lymphocytes, hematopoietic stem cells (HSCs) are isolated from the patient’s bone marrow, transduced ex vivo using a gamma retroviral vector carrying cDNA of the enzyme adenosine deaminase, and returned to the patient’s blood. As a result, all HSC progeny, including lymphocytes, carry transgenes encoding the normal enzyme. A similar method has been proposed for the treatment of X-linked adrenoleukodystrophy caused by an *ABCD1* gene mutation (adenosine-triphosphate-binding cassette transporter). After the transplantation of autologous HSCs, where the mutant gene has been corrected using a lentiviral vector, the patient regained phagocyte function, including the restoration of microglia cell function, which enables normal fatty acid metabolism in the CNS [12].

In practical medicine, allogeneic HSC transplantation is the most widely used approach for the treatment of malignant and benign diseases of the hematopoietic system in clinical care [13,14]. For cell therapy, HSCs are derived from umbilical cord blood, peripheral blood, and bone marrow [15]. Umbilical cord blood mononuclear fraction contains HSCs, progenitor endothelial cells [16], multipotent mesenchymal stromal cells [17], and other even less differentiated stem cells with pluripotent properties [18,19,20], which gives reason to consider them as a potential source for cell therapy (in autografting and allografting) for ischemic, traumatic, and degenerative diseases [21,22,23]. In addition, UCB-MCs produce antioxidant, angiogenic, and neurotrophic factors, which can also have a stimulating effect on the regeneration of the target organ [24,25,26,27].

Currently, umbilical cord blood mononuclear cells (UCB-MCs) are being actively tested in the treatment of CNS disorders. It is important to note that UCB-MCs may be used for transplantation without HLA matching and immunosuppression therapy. The UCB-MC population mostly consists of immature T-cells with a higher CD4+/CD8+ T-cell ratio [28]. The biosafety of the allogenic transplantation of UCB-MCs was demonstrated in the treatment of patients with non-hematopoietic degenerative conditions [21]. The beneficial effects of UCB-MCs were shown in the aged brain [29] and in the treatment of Parkinson’s disease [30], amyotrophic lateral sclerosis (ALS) [31,32,33], ischemic stroke [34,35], and neurotrauma [36,37]. The limited number of available cells from a single donor remains a serious problem when using UCB-MCs in clinical practice. Hence, genetic modification of UCB-MCs can increase their therapeutic potential, enabling their use in targeted pathogenetic therapy. Genetically engineered UCB-MCs can migrate to the site of degeneration and enable the local and temporary production of recombinant therapeutic molecules. In earlier studies, we demonstrated the positive effect of gene-modified UCB-MCs producing recombinant vascular endothelial growth factor (VEGF) on the symptomatic outcome and life-span of transgenic ALS mice [38].

The link between ALS and VEGF, which is involved in the survival of motor neurons, has been shown in in vitro and in vivo experiments [39]. The important role of VEGF in embryogenesis as an angiogenic and neurogenic factor suggests the potential use of recombinant VEGF to modulate neuroplasticity in various CNS diseases [40]. The VEGF family includes VEGF-A (previously simply known as VEGF), VEGF-B, VEGF-C, VEGF-D, VEGF-E, and placental growth factor (PlGF) [41]. As a result of alternative splicing of the gene encoding VEGF-A, molecules consisting of 121, 145, 165, 189, or 206 amino acids are synthesized. Mostly soluble VEGF121 (diffusing over long distances) and VEGF165 (reaching distant and nearby targets) are the focus of current intensive research [42]. Preclinical studies have demonstrated the beneficial effects of VEGF in the treatment of neurological disorders [40]. The neuroprotective effect of VEGF on the brain was shown in ischemic stroke [43,44], traumatic brain injuries [45,46,47], spinal cord injuries [48,49], and neurodegenerative diseases [42,50,51].

Thus, ex vivo gene modification of UCB-MCs allows us to enhance their native neuroprotective properties. In addition, this approach can be useful to obtain UCB-MCs with the therapeutic effects required for the treatment of human diseases based on the temporal synthesis and secretion of specific bioactive therapeutic molecules responsible for the correction of a particular pathological disorder. We have recently demonstrated the positive effect of UCB-MCs transduced with Ad5-LTF carrying the human lactoferrin gene on the recovery of maxillofacial phlegmon in rats [52] and the induction of angiogenesis by UCB-MCs transduced with Ad5-VEGF165 applied in an in vivo Matrigel plug assay [53]. However, the biosafety of UCB-MC transduction using an adenoviral vector and transgene overexpression on the native functional characteristics of UCB-MCs remains unclear. Therefore, in this study we assessed the transcriptome landscape and cytokine profiling of genetically modified human UCB-MCs transduced with an adenoviral vector (Ad5) carrying a cDNA encoding human VEGF165.

## 2. Materials and Methods

### 2.1. Study Design

In continuing research to develop an effective gene therapy approach to stimulate regeneration in the CNS, we have demonstrated the positive effect of genetically modified UCB-MCs in transgenic ALS mice [38] and in rats with spinal cord injuries [54] and stroke [55]. Following these reports, we expanded this study to estimate the biosafety of UCB-MCs transduced with Ad5-VEGF165 in vitro. In a preliminary independent experiment using cord blood samples (*n* = 3) we assessed the efficacy of UCB-MC transduction with Ad5-VEGF165 and Ad5-GFP at MOI = 10. The synthesis of mRNA transgenes and recombinant proteins (VEGF and GFP) was confirmed by RT-PCR, Western blot, ELISA, flow cytometry, and fluorescence microscopy. The main goal of this study was to assess the impact of an adenoviral vector (Ad5) and a transgene (*vegf165*) on the transcription and secretion patterns of genetically modified UCB-MCs derived from six cord blood samples using RNA-seq and a multiplex assay, respectively.

### 2.2. Preparation of Umbilical Cord Blood Mononuclear Cells

Umbilical cord blood was collected after informed consent had been obtained from the pregnant women, and prenatal testing to determine their eligibility for blood donation was carried out. The cord blood was collected in containers with citrate, phosphate, dextrose, and adenine (CDFA-1) (Baxter International Inc., Deerfield, IL, USA) in accordance with the protocol that adheres to the legitimate and ethical standards generally accepted in the stem cell bank of Kazan State Medical University and approved by the Kazan State Medical University Animal Care and Use Committee (approval No. 5 dated 26 May 2020). Over the next 12 h, umbilical cord blood mononuclear cells (UCB-MCs) were isolated by standard density barrier sedimentation using Ficoll (1.077 g/mL), as described previously [38].

### 2.3. Adenoviral Transduction of UCB-MCs

Recombinant serotype 5 (Ad5) adenoviral vectors carrying cDNA of the human VEGF gene and a reporter gene encoding green fluorescent protein (GFP) were generated using Gateway cloning technology according to manufacturer’s instructions (Invitrogen, Carlsbad, CA, USA), as described previously [38]. The titres of Ad5-VEGF165 (2.6 × 10^9^ PFU/mL) and Ad5-GFP (1.2 × 10^10^ PFU/mL) were determined by plaque formation assay in HEK-293 cells (ATCC, 293T/17 [HEK 293T/17] CRL-11268TM) [56]. Genetic modification of UCB-MCs with Ad5-VEGF165 or Ad5-GFP was performed with a multiplicity of infection (MOI) equal to 10 (MOI = 10) according to the UCB-MC count and Ad5 titre. The samples of gene-modified and native UCB-MCs and conditioned culture medium were analysed 72 h after incubation in an RPMI-1640 medium (PanEco, Moscow, Russia) supplemented with 10% FBS and a mixture of antibiotic penicillin and streptomycin (100 U/mL and 100 μg/mL, respectively) at 37 °C under 5% CO_2_. All the work with cell cultures was performed under aseptic conditions in a Herasafe biological safety cabinet (Germany) with respect to the generally accepted rules of work with eukaryotic cells.

### 2.4. Flow Cytometry and Fluorescence Microscopy

The efficiency of UCB-MC transduction with Ad5 carrying a reporter gene encoding green fluorescent protein (Ad5-GFP) was analysed 72 h after cell transduction. The synthesis of GFP in gene-modified cells was examined using an Axio Observer Z1 inverted fluorescence microscope (Carl Zeiss, Oberkochen, Germany). The number of GFP-positive UCB-MC+Ad5-GFP was estimated using a BD FACSAria III flow cytomography fluorimeter (BD Bioscience, New York, NY, USA) and BD FACS Diva7 software (BD Bioscience, New York, NY, USA) [57].

### 2.5. Quantitative Reverse Transcription PCR

The mRNA level of VEGF165 and GFP in UCB-MC+Ad5-VEGF and UCB-MC+Ad5-GFP was revealed using qRT-PCR. The total RNA was isolated from gene-modified UCB-MCs 72 h after transduction using the TRIzol reagent (Thermo Fisher Scientific, Waltham, MA, USA) and cDNA synthesis was performed. qRT-PCR was conducted on the Real-Time CFX96 Touch instrument (BioRad Laboratories, Hercules, CA, USA). The primer and probe sequences used in qRT-PCR are listed in Table 1. Triplicate reactions were performed for each sample, and the ΔΔCt (Livak) method was used to calculate the average relative target gene expression normalized by β-actin rRNA [58]. Standard curves were generated using serial dilutions of plasmid DNA containing the respective inserts (VEGF and GFP). The target gene mRNA levels in native UCB-MCs were taken as 100%.

### 2.6. Western Blotting

The ability of UCB-MC+Ad5-VEGF to synthetize recombinant VEGF was studied 72 h after the transduction of UCB-MCs with Ad5-VEGF165 using Western blot. The protein extracts obtained from native and genetically modified UCB-MCs were separated by electrophoresis in a 15% polyacrylamide gel in the presence of sodium dodecyl sulphate (SDS-PAGE) and were transferred onto PVDF (Polyvinylidene difluoride) membranes. The non-specific binding of primary antibodies (Abs) was blocked using 5% non-fat milk diluted in Twin-PBS (pH 7.4) at 21 °C for 4 h.

Afterwards, the PVDF membranes were incubated with Abs against VEGF (Sigma, Saint Louis, MO, USA, V6627, 1:1000) and β-actin (Genscript, Piscataway, NJ, USA, A00730-40, 1:3000) overnight at 4 °C. Horseradish peroxidase conjugated Abs were used to visualize the target proteins [59]. The data obtained on UCB-MC+Ad5-GFP and native UCB-MCs were used for comparative analysis. Two independent experiments were performed in order to obtain the results.

### 2.7. Enzyme-Linked Immunosorbent Assay

The potential of UCB-MC+Ad5-VEGF165 to secrete recombinant VEGF was investigated in supernatants collected 72 h after the incubation of gene-modified and native UCB-MCs, using an enzyme-linked immunosorbent assay (ELISA) [60] and ELISA kit for human VEGF (DuoSet, DY293B). The levels of soluble VEGF, according to the optical density, was measured using a BioRad xMark multifunctional microplate spectrophotometer (BioRad, Hercules, CA, USA) at a wavelength of 450 nm. The standard curves plotted using serial dilutions of the recombinant protein provided in the kit were used for quantification. The results were obtained from two technical repetitions.

### 2.8. Multiplex Secretome Profiling

Supernatants obtained 72 h after the incubation of native and gene-modified UCB-MCs (UCB-MC+Ad5-VEGF165 and UCB-MC+Ad5-GFP) that were prepared from six individual donors were used for cytokine, chemokine, and growth factor analysis with commercially available fluorophore-conjugated microspheres (fluorophore-conjugated beads), employing xMap technology (Luminex, Austin, TX, USA) [61]. Bio-Plex Pro™ Human Cytokine Screening 48-Plex was used in this study. Each sample was studied in triplicate. Standard curves for each cytokine were generated using standards provided by the manufacturer. The data collected was analysed using MasterPlex CT control software v.3 and MasterPlex QT analysis software v.3 (MiraiBio, San Bruno, CA, USA).

### 2.9. Transcriptome Sequencing and Bioinformatics Analysis

Whole Transcriptome Sequencing “WTS” of native UCB-MCs and gene-modified UCB-MCs (UCB-MC+Ad5-VEGF165 and UCB-MC+Ad5-GFP) obtained from six individual donors was performed using the Illumina platform [62]. Total RNA was extracted 72 h after the incubation of UCB-MCs using TRIzol (Thermo Fisher Scientific) treated with DNase I and purified using the QIAGEN RNeasy Mini Kit. The Agilent Bioanalyzer 2100 and Qubit (Thermo Fisher Scientific) were used to assess the quality and concentration of the isolated RNA samples. All the RNA samples had RNA integrity numbers of more than 8.10. The target mRNA samples were enriched from previously isolated and characterized total RNA using the NEBNext Poly(A) mRNA Magnetic Isolation Module (NEB, #E7490 S) kit (New England Biolabs, Ipswich, MA, USA). cDNA libraries from all the mRNA samples were prepared using the NEBNext Ultra II Directional RNA library prep kit and sample purification beads (NEB, #E7765 S) (New England Biolabs). The DNA sequence of each cluster in flow cells was determined in 150 cycles according to Sequencing By Synthesis (SBS) technology, employing the NextSeq 500/550 High Output v2.5 Kit (150 cycles) and the NextSeq500 Sequencing System (Illumina, San Diego, CA, USA) using the 2 × 75 bp mode. After evaluating the quality of the sequencing, the obtained reads were aligned with the human reference transcriptome assembly GRCh38 (hg38) from Genome Reference Consortium (GCA_000001405.15 GCF_000001405.26) using a Kallisto pseudoaligner [63]. Differentially expressed transcripts and genes were annotated using the R “sleuth” package (www.r-project.org, www.rdocumentation.org/packages/sleuth) (accessed date on 17 November 2022). A functional enrichment analysis of the genes with >10 TPM (transcripts per million) was performed using WebGestalt (WEB-based Gene Set Analysis Toolkit) [64].

### 2.10. Statistics

The statistical analysis was performed using GraphPad Prism^®^ 7 software (GraphPad, Inc., La Jolla, CA, USA). The data are presented as the mean ± standard error (SE). Statistically significant differences were assessed using a one-way analysis of variance (ANOVA) followed by Tukey’s test. Statistical significance is denoted by *p* < 0.05.

## 3. Results

### 3.1. Transduction Efficacy and Expression of Transgenes in UCB-MCs

**Figure 1:**
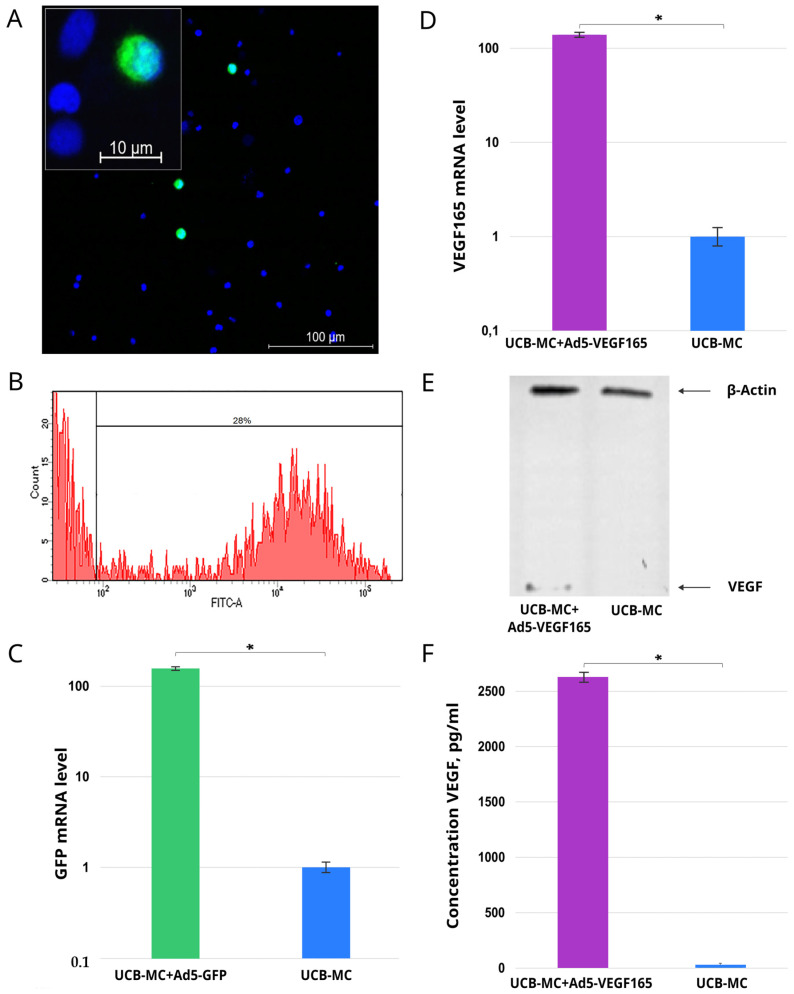
Expression of reporter green fluorescent protein (GFP) gene and recombinant gene encoding vascular endothelial growth factor (*vegf165*) by genetically modified human umbilical cord blood mononuclear cells (UCB-MCs) 72 h after transduction with Ad5-GFP and with Ad5-VEGF165, respectively. (**A**) Fluorescence microscopy demonstrates GFP-positive UCB-MC+Ad5-GFP (green glow). Nuclei were stained with Hoechst 33342 (blue glow). (**B**) Flow cytometry revealed 28% of UCB-MCs synthetizing GFP. (**C**) Quantitative analysis of GFP mRNA levels in UCB-MC+Ad5-GFP by qRT-PCR. (**D**) Quantitative analysis of VEGF165 mRNA levels in UCB-MC+Ad5-VEGF165 using qRT-PCR. (**E**) Western blotting analysis of recombinant VEGF165 in UCB-MC+Ad5-VEGF165. (**F**) Content of the recombinant VEGF165 in the conditioned culture medium after the incubation of UCB-MC+Ad5-VEGF165 using ELISA. *—*p* < 0.05.

This part of the study was conducted as a preliminary task using distinct cord blood samples (*n* = 3). Using fluorescence microscopy in UCB-MCs transduced with Ad5-GFP, a specific green glow was detected in the cytoplasm of the gene-modified cells (Figure 1A). The flow cytometry study revealed 28.0 ± 2.3% of the GFP-positive cells (Figure 1B). The qRT-PCR analysis demonstrated an increase in GFP mRNA by 100-fold in UCB-MC+Ad5-GFP when compared with non-transduced UCB-MCs (Figure 1C). The analysis of recombinant *vegf165* expression in the UCB-MC+Ad5-VEGF165 also revealed a 100-fold elevated level of mRNA (Figure 1D). The synthesis of VEGF165 in gene-modified UCB-MCs was confirmed by Western blot analysis and showed bands corresponding to a positive reaction with Abs against VEGF (Figure 1E). Secretion of the recombinant protein was documented using ELISA, which detected 2623.0 ± 45.5 pg/mL of VEGF in the conditioned culture medium after UCB-MC+Ad5-VEGF165 incubation, compared with native UCB-MCs (22.1 ± 2.1 pg/mL) (Figure 1F).

### 3.2. Transcriptome Analysis of the Genetically Modified UCB-MCs

**Figure 2:**
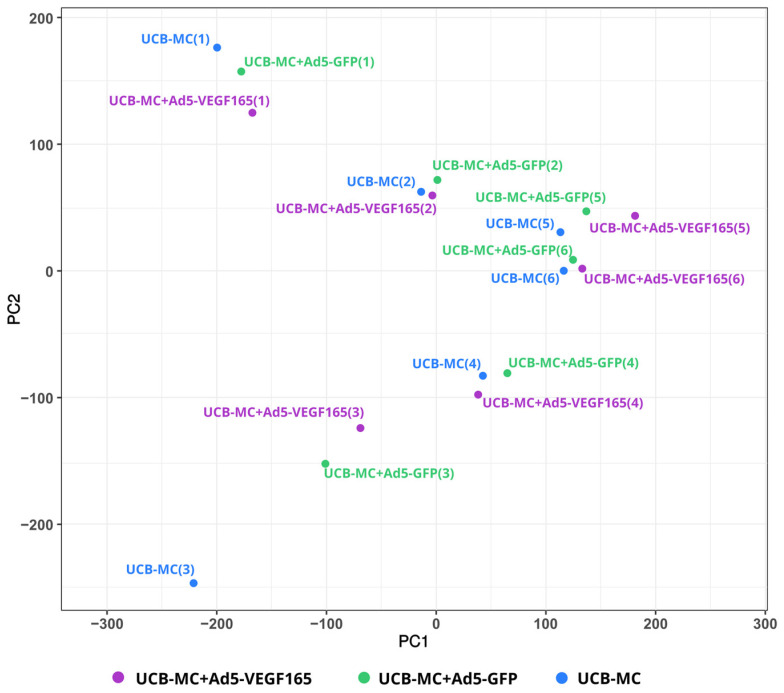
Principal component analysis (PCA) of gene expression data in gene-modified UCB-MCs (UCB-MC+Ad5-VEGF165 and UCB-MC+Ad5-GFP) and native UCB-MCs 72 h after incubation. The samples obtained from six individual donors are presented as numbers in parentheses (1–6).

**Figure 3:**
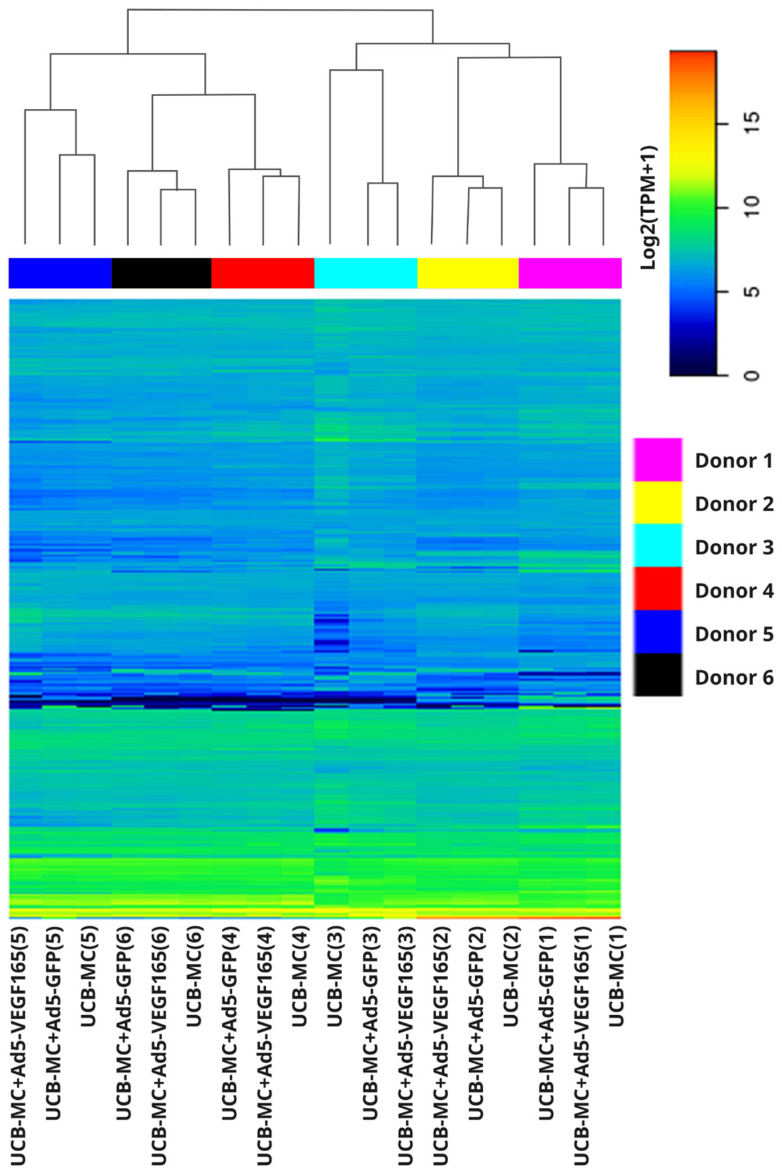
Heatmap representing log2 transcripts per million (TPM) in gene-modified UCB-MCs (UCB-MC+Ad5-VEGF165 and UCB-MC+Ad5-GFP) and native UCB-MCs 72 h after incubation. Data is presented for the first 1760 genes whose expression was at least 100 transcripts per million. The samples obtained from six individual donors are presented as numbers in parentheses (1–6).

**Figure 4:**
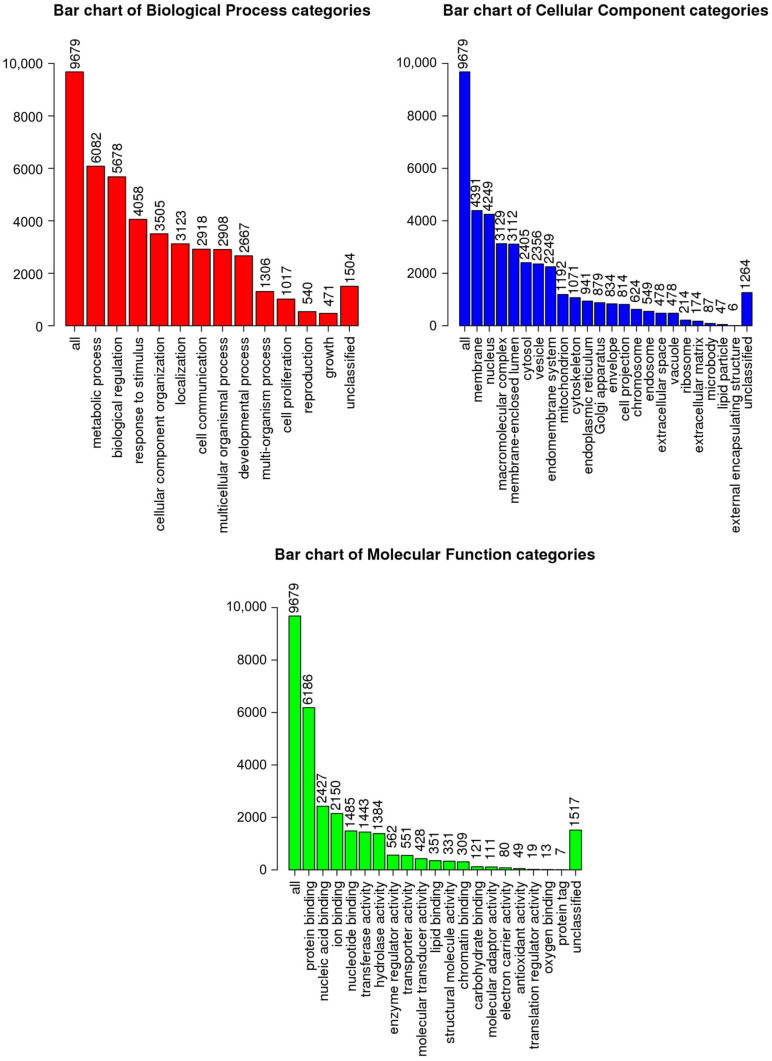
Gene Ontology (GO)-based enrichment analysis of native UCB-MC transcriptomes. GO terms are presented for the categories of biological processes, cellular components, and molecular functions.

A comparative analysis of the mRNA transcription profiles in native UCB-MCs and gene-modified UCB-MCs (UCB-MC+Ad5-GFP and UCB-MC+Ad5-VEGF165) obtained from six individual samples of cord blood (donors) was performed based on 18 cDNA libraries. A bioinformatics analysis of the RNA-seq data revealed 2.4–2.8 × 10^7^ paired reads per samples and transcripts of 10164 genes. The principal component analysis (PCA) of the RNA-seq data showed that samples representing the three comparison groups did not cluster together. However, the samples were grouped according to the cord blood source (donor). The results obtained from the principal component analysis are visualized on a biplot (Figure 2). Among a wide range of genes with an expression of at least 100 transcripts per million, there were no differences in the transcriptome profiles of the native and gene-modified UCB-MCs (Figure 3). At the same time, the recombinant genes *gfp* (log2(Fold change) = 7.15, q value < 0.05) and *vegf165* (log2(Fold change) = 4.41, q value < 0.05) showed increased expression in UCB-MC+Ad5-GFP and UCB-MC+Ad5-VEGF165, respectively, compared to the non-transduced UCB-MCs, as expected. Functional profiling of the detected genes was performed using a GO-based enrichment analysis, where genes whose representation was above 10 TPM in at least one of the samples studied were included. Interpretation using WebGestalt software (http://www.webgestalt.org) allowed the formation of a functional profile of native and gene-modified UCB-MCs, including three groups of annotations (biological processes, molecular functions, and cellular components) presented in the highest category of the Gene Ontology hierarchy. The findings showed that the majority of the genes associated with biological processes were related to metabolism. In the category of cellular components, the majority of the genes detected were related to the cell membrane and cell nucleus, and in the category of molecular functions, they were related to protein binding (Figure 4).

### 3.3. Secretome Profiling of the Genetically Modified UCB-MCs

**Figure 5:**
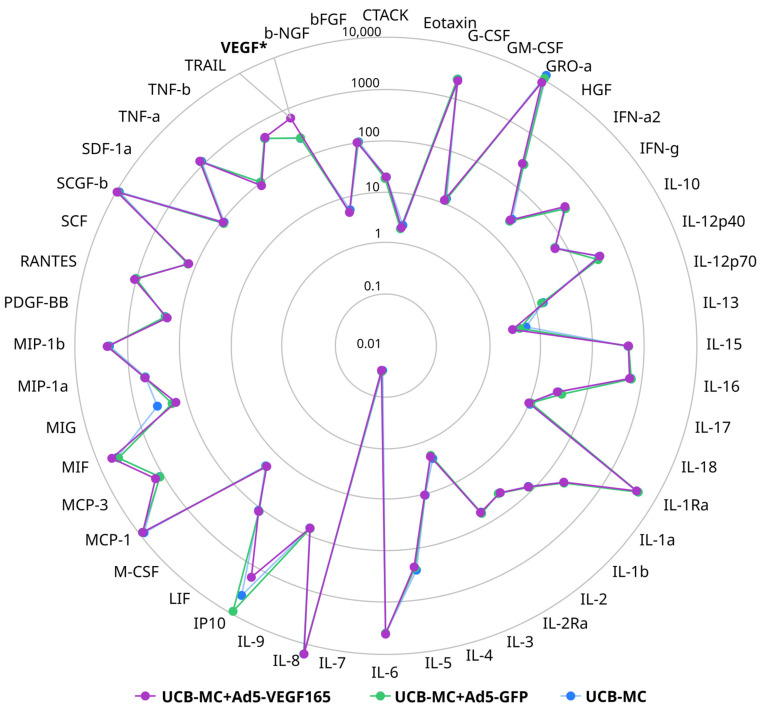
Radial comparative diagram of cytokine, chemokine, and growth factor representation in supernatant obtained 72 h after the incubation of gene-modified UCB-MCs (UCB-MC+Ad5-VEGF165 and UCB-MC+Ad5-GFP) and native UCB-MCs. The concentration of the analytes studied is presented in pg/mL. *—*p* < 0.05.

The multiplex secretome analysis of cytokines, chemokines, and growth factors, including 48 analytes in supernatants obtained 72 h after the incubation of native and gene-modified UCB-MCs (UCB-MC+Ad5-VEGF165 and UCB-MC+Ad5-GFP) prepared from six cord blood samples, did not reveal any differences between the groups studied. However, in UCB-MC+Ad5-VEGF165, an increase in VEGF content was observed when compared with native UCB-MCs and UCB-MC+Ad5-GFP (Figure 5).

**Figure 6:**
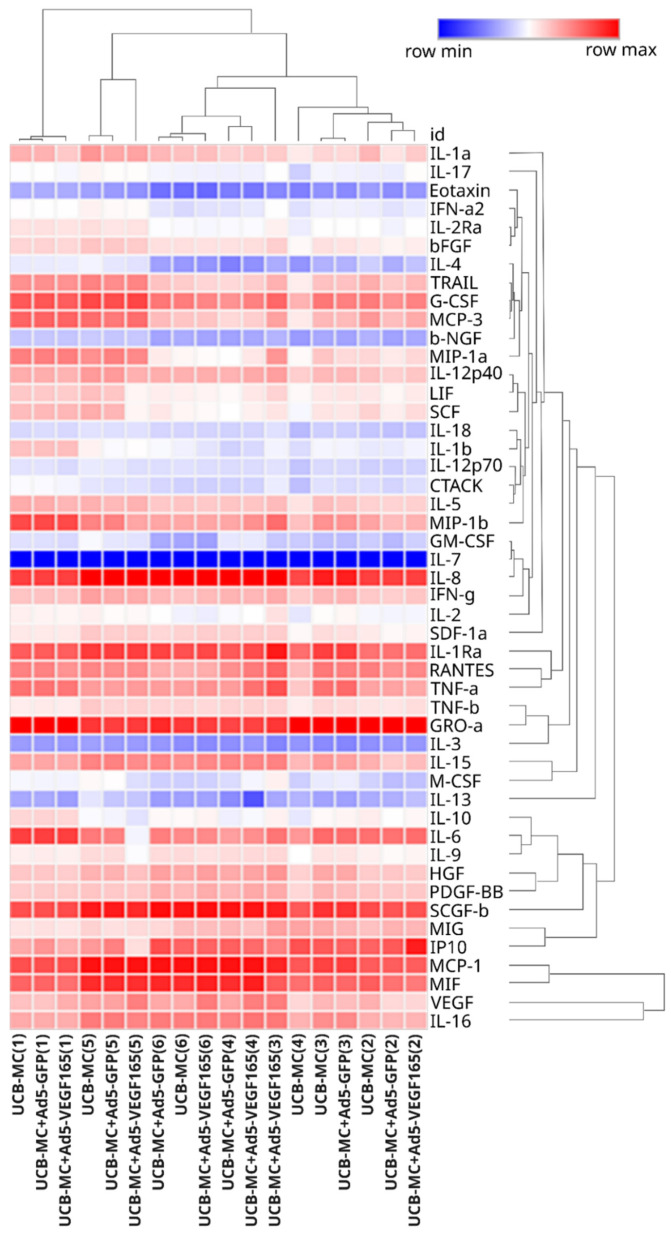
Heatmap of cytokine, chemokine, and growth factor representation in supernatant obtained 72 h after the incubation of gene-modified UCB-MCs (UCB-MC+Ad5-VEGF165 and UCB-MC+Ad5-GFP) and native UCB-MCs. The samples obtained from six individual donors are presented as numbers in parentheses (1–6).

The cluster analysis of the obtained secretomes also confirmed that the genetic modification of UCB-MCs does not affect their secretion of cytokines, chemokines, and growth factors (Figure 6). At the same time, the samples of UCB-MCs obtained from individual donors had different secretion profiles of the analytes studied, and the grouping of gene-modified cells was consistent with the UCB-MC source.

Thus, UCB-MC transduction with Ad5-VEGF165 and Ad5-GFP at MOI = 10 revealed effective expression of the transgenes at mRNA and protein levels. The bioinformatics analysis of the RNA-seq data obtained from the native UCB-MCs and gene-modified UCB-MCs (UCB-MC+Ad5-VEGF165 and UCB-MC+Ad5-GFP) revealed that the adenoviral construct (Ad5) or transgene (*vegf165*) had no effect on 10164 gene transcripts. The multiplex secretome analysis of 48 cytokines, chemokines, and growth factors also showed no differences in the secretion patterns of the native and genetically modified UCB-MCs and was in line with the results obtained from the bioinformatics analysis of the UCB-MC transcriptomes.

## 4. Discussion

In recent years, the steady growth of gene therapy research has demonstrated its significant impact on regenerative therapies. Gene therapy is considered a powerful tool not only for correcting the function of a mutated gene, but also for targeting changes in cell function [65]. Despite the great promise of gene therapy for the treatment of human diseases, the risk of side effects is one of the main reasons for the slow introduction of a drug containing recombinant cDNA as a pharmaceutical product. Therefore, biosafety aspects play a crucial role in the translation of a gene therapy drug from biomedical research into clinical care [66].

Gene therapy implies the delivery of therapeutic genes into the recipient’s body using non-viral or viral vectors (direct gene therapy) or on cell carriers (cell-mediated gene therapy). The advantages and disadvantages of each method of transgene delivery have been discussed in many research publications concerning gene therapy [3,67]. The choice of the vector or cell delivery system for the therapeutic gene may depend on the nature of the disease (inherited or acquired), the duration of the planned treatment (lifetime or in the acute or chronic phase of the disease), the assumed effect (local or systemic), and the intended effect (etiotropic, pathogenetic, or symptomatic).

An important focus of gene therapy is to increase the level of expression of genes encoding trophic factors and growth factors that stimulate the regenerative potential of organs associated with the disease. The method of delivery of therapeutic genes encoding biologically active molecules using ex vivo gene-modified cells, which serve as producers of these molecules, serves as an alternative to the intravenous administration of recombinant proteins. A significant disadvantage of using recombinant proteins for replacement therapy is their short half-life and the need for the repeated use of expensive drugs during the course of disease treatment. Transplantation of the genetically modified cells expressing transgenes is reasonable, not only through the production of therapeutic molecules, but also through the effect of transgene carrier cells (stem cells, progenitor cells, or mature cells) on the regenerative capacity of the damaged tissue.

In this regard, an attractive opportunity in regenerative medicine is the use of UCB-MCs to deliver recombinant genes encoding growth and trophic factors. Numerous studies have demonstrated the feasibility of using UCB-MCs, not only to correct haematological disorders, but also to stimulate the regeneration of various tissues and organs in ischemic and degenerative diseases [21]. Cord blood cells are readily available and have the lowest immunogenicity compared to other allogeneic cells [28,68]. Also of interest is the legal and ethical opportunity to apply UCB-MCs in clinical practice.

In the therapy of ischemic diseases, genetically modified UCB-MCs are used to stimulate angiogenesis. A positive therapeutic effect was achieved in an animal model of chronic hind limb skeletal muscle ischemia after the transplantation of UCB-MCs overexpressing human VEGF [69]. In a rat model of myocardial infarction, UCB isolated HSCs overexpressing VEGF and PDGF (platelet-derived growth factor gene) [70], or VEGF and angiopoietin-1 (Ang1) genes [71] inhibited the development of cardiac muscle necrosis and increased capillary density in the myocardium. In our studies, in order to stimulate regeneration in the CNS, we developed UCB-MCs simultaneously overexpressing three recombinant neuroprotective factors (VEGF, GDNF [glial-cell-line-derived neurotrophic factor], and NCAM [neural cell adhesion molecule]) [55,72,73].

The successful translation of a gene-cell pharmaceutical product into clinical practice requires the establishment of its efficacy and the conduct of preclinical studies to ensure its biosafety. The genetic modification of cells using a plasmid or viral vector, through the influence of the vector itself or its expressed products, can change the genotype and/or phenotype of genetically modified cells. In the present study, we studied the transcriptome and secretome patterns of UCB-MCs transduced with Ad5 carrying cDNA of human *vegf165* and compared them with UCB-MCs transduced with Ad5-GFP and native UCB-MCs. Preliminary molecular and cellular analyses confirmed the efficacy of UCB-MC transduction with a human adenovirus serotype 5 (Ad5) vector. At an MOI of 10, the number of UCB-MC+Ad5-GFP was 28% and the recombinant *vegf165* mRNA level in UCB-MC+Ad5-VEGF165 was 100-fold higher than in native UCB-MCs. The synthesis and secretion of recombinant VEGF in UCB-MC+Ad5-VEGF165 were also established by Western blot and ELISA, respectively.

The bioinformatics analysis of the native and genetically modified UCB-MC RNA-seq data revealed that the variability observed in the transcriptome is primarily attributed to individual donor variability rather than the genetic modification of UCB-MC transcriptomes. The three annotation groups (biological processes, molecular functions, and cellular components) presented in the highest category of the Gene Ontology hierarchy showed similar GO-based enrichment patterns in modified and native cells, which was consistent with the absence of differences in the transcriptomes. The secretome profiling (the secretion of studied cytokines, chemokines, and growth factors) of UCB-MCs did not reveal differences between native and gene-modified UCB-MCs (UCB-MC+Ad5-VEGF165 and UCB-MC+Ad5-GFP). Consequently, we found no negative effects of the adenoviral vector (the study of UCB-MC+Ad5-GFP) or transgene (*vegf165*) expression (the study of UCB-MC+Ad5-VEGF165) on the transcription activity and functional status of gene-modified UCB-MCs. The increased *vegf165* expression in UCB-MC+Ad5-VEGF165, demonstrated by transcriptome and secretome analyses, aligns with the results obtained by qRT-PCR, Western blot, and ELISA in the preliminary study. At the same time, it is important to note that transcriptomes and secretomes differed between the UCB-MC samples obtained from individual donors.

Thus, UCB-MC+Ad5-VEGF165 retain their native properties and actively secrete recombinant VEGF. These data support the rationality of using genetically modified UCB-MCs for the temporary synthesis and secretion of recombinant therapeutic molecules in the treatment of neurological diseases. Before translating gene therapy with UCB-MCs into clinical trials, more research is needed to develop a GMP protocol for the preparation of genetically engineered UCB-MCs and to study the biosafety, dosage, transplantation methods, targeting, and pharmacokinetics of recombinant molecules in large animals with morphological, functional, and biochemical characteristics similar to humans.

We believe that the results of this work provide a solid fundamental platform for biosafety research into genetically modified UCB-MCs and will enable the translation of cell-mediated gene therapies into clinical care.

## 5. Conclusions

The safety of pharmaceutical products containing artificial genetic material is one of the important issues when implementing gene therapy in practical medicine. Two modes of introducing transgenes into the patient, including virus-mediated and cell-mediated delivery, are currently being actively investigated. These approaches have advantages and disadvantages in terms of transgene expression efficiency and biosafety. The aim of this research was to investigate the biosafety of genetically modified UCB-MCs transduced with a human adenovirus serotype 5 (Ad5) vector carrying cDNA encoding human VEGF. The efficacy of *vegf165* expression has been proven at the level of mRNA transcription, recombinant protein synthesis, and secretion. It is worth noting that the efficient production of recombinant VEGF165 does not negatively affect the transcriptome profile and secretion of the studied cytokines, chemokines, and growth factors by genetically modified UCB-MCs. At the same time, the UCB-MC samples obtained from six donors had different transcriptome and secretion patterns, indicating individual variability. Thus, we propose that the data on effective transgene expression and preservation of the native properties of genetically modified UCB-MCs brings us closer to the possibility of using UCB-MCs as cell carriers of artificial genetic materials and as producers of recombinant therapeutic molecules in ex vivo gene therapy.

## Figures and Tables

**Table 1:** Primer sequences used for real-time quantitative reverse transcriptase polymerase chain reaction (qRT-PCR).

Name	Nucleotide Sequence
β-actin-TM-Forward (human)	GCGAGAAGATGACCCAGGATC
β-actin-TM-Reverse (human)	CCAGTGGTACGGCCAGAGG
β-actin-TM-Probe (human)	[FAM]CCAGCCATGTACGTTGCTATCCAGGC[BH1]
hVEGF-TM49-Forward (human)	TACCTCCACCATGCCAAGTG
hVEGF-TM110-Reverse (human)	TGATTCTGCCCTCCTCCTTCT
hVEGF-TM-Probe (human)	[FAM]TCCCAGGCTGCACCCATGG[BH1]
GFP-TM-Forward	AGCAAAGACCCCAACGAGAA
GFP-TM-Reverse	GGCGGCGGTCACGAA
GFP-TM-Probe	[FAM]CGCGATCACATGGTCCTGCTGG[BH1]

## Data Availability

The data presented in this study is available on request from the corresponding author.
